# Humoral immunity for durable control of SARS-CoV-2 and its variants

**DOI:** 10.1186/s41232-023-00255-9

**Published:** 2023-01-12

**Authors:** Ryutaro Kotaki, Saya Moriyama, Yoshimasa Takahashi

**Affiliations:** Research Center for Drug and Vaccine Development, National Institute of Infectious Diseases, Tokyo, 162-8640 Japan

**Keywords:** Immunological memory, Vaccine, B cell, Antibody, SARS-CoV-2

## Abstract

The coronavirus disease 2019 (COVID-19) pandemic is ongoing because of the repeated emergence of severe acute respiratory syndrome coronavirus 2 (SARS-CoV-2) variants, highlighting the importance of developing vaccines for variants that may continue to emerge. In the present review, we discuss humoral immune responses against SARS-CoV-2 with a focus on the antibody breadth to the variants. Recent studies have revealed that the temporal maturation of humoral immunity improves the antibody potency and breadth to the variants after infection or vaccination. Repeated vaccination or infection further accelerates the expansion of the antibody breadth. Memory B cells play a central role in this phenomenon, as the reactivity of the B-cell antigen receptor (BCR) on memory B cells is a key determinant of the antibody potency and breadth recalled upon vaccination or infection. The evolution of memory B cells remarkably improves the reactivity of BCR to antigenically distinct Omicron variants, to which the host has never been exposed. Thus, the evolution of memory B cells toward the variants constitutes an immunological basis for the durable and broad control of SARS-CoV-2 variants.

## Background

Severe acute respiratory syndrome coronavirus 2 (SARS-CoV-2), the causative virus of coronavirus disease 2019 (COVID-19), is currently threatening the lives of people worldwide. COVID-19 presents with mild to severe respiratory symptoms, and more than 500 million cases with more than 6 million deaths have been reported globally as of August 31, 2022 (https://covid19.who.int/). Since the initial case was identified in December 2019, several vaccines that confer protection against SARS-CoV-2 have rapidly been developed and authorized for emergency use. COVID-19 vaccines efficiently confer protection against SARS-CoV-2 infection, hospitalization, and death [1, 2].

Immunological memory responses that are formed and maintained in response to primary antigen exposure protect the host from subsequent viral infection. Immunological memory responses are of particular importance for vaccination, as they are the basis for conferring protection against re-infection by a variety of pathogens [3]. Immunological memory involves two classes of responses, humoral and cellular immunity, driven by B and T cells, respectively. The present review will focus on the humoral immune system, while cellular immunity to SARS-CoV-2 has been reviewed elsewhere [4].

**Fig. 1:**
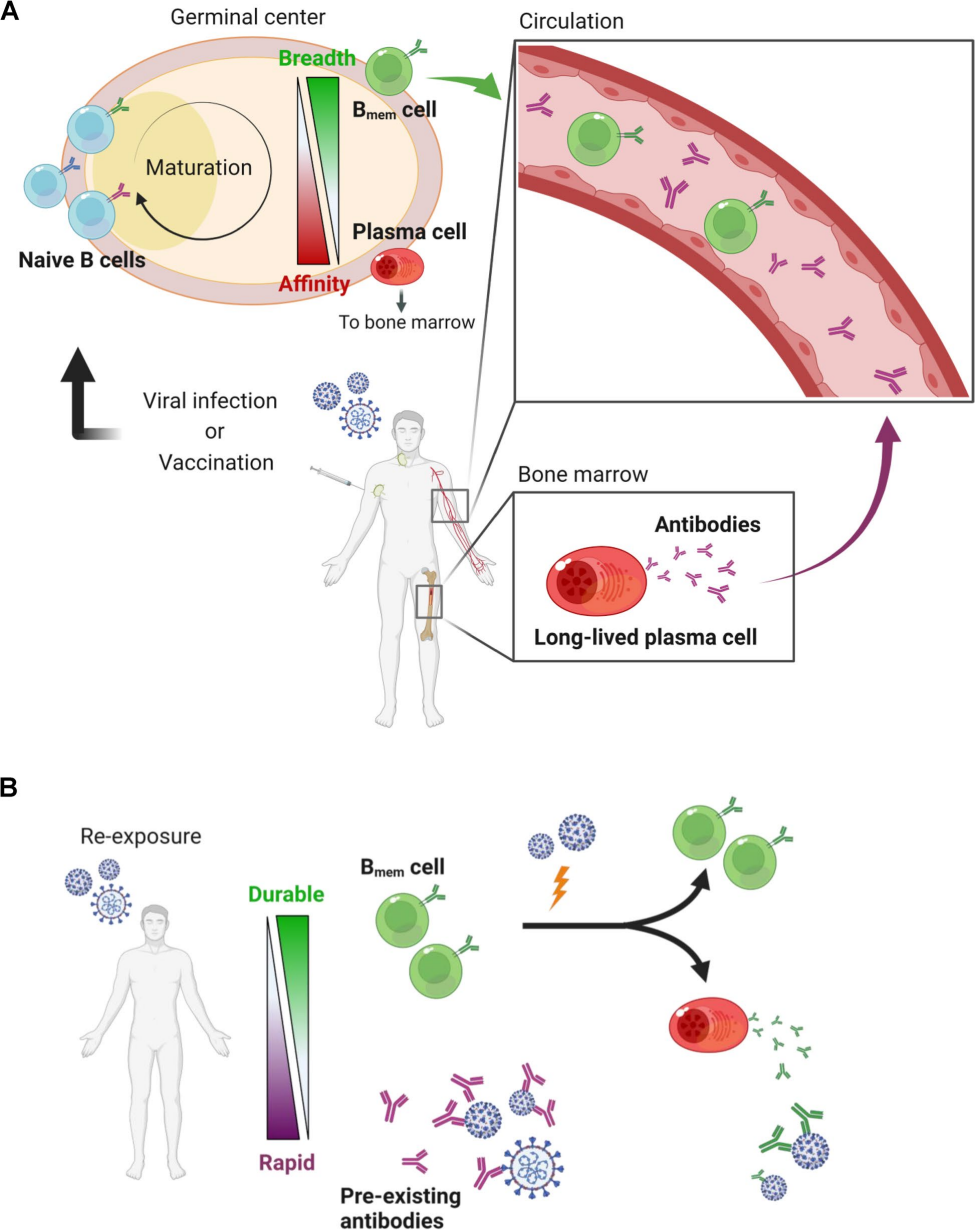
Humoral immune memory responses. **A** Humoral immune responses to a primary antigen exposure are depicted. Viral infection or vaccination induces B_mem_ and plasma cells through germinal center reaction, conferring immunological memory. **B** Memory responses to antigen re-exposure are described. Pre-existing antibodies secreted from long-lived plasma cells rapidly eliminate the viruses, whereas B_mem_ cells function as a backup reservoir for plasma cells. The figure was created with BioRender.com

Humoral memory responses to secondary or further antigen exposure involve two defense walls: long-lived plasma and memory B (B_mem_) cells. Both long-lived B cells largely develop within germinal centers (GCs), where those with high-affinity B cell receptors (BCRs) to the antigens emerge with somatic hypermutations and are clonally selected (Fig. 1A). However, the two long-lived B cells are functionally different from each other: plasma cells mediate prompt and effective protection via pre-existing antibodies as the first line of defense, and B_mem_ cells serve as the backup of pre-existing antibodies by robustly supplying plasma cells in response to re-invading antigens (Fig. 1B). In cases of acute viral infection, the circulating antibody level declines in a biphasic manner after its peak: a rapid decline in the early phase (< 3 months) followed by a gradual decline in the late phase (< 1 year) [5]. The kinetics are compatible with that of plasma cells in the bone marrow, which decline from its peak to the baseline within 1 year [5]. By stark contrast, B_mem_ cells, circulating in the peripheral blood, increase in numbers in 2–3 weeks and are stably maintained for up to 1 year [6, 7]. Further differences between plasma cells and B_mem_ cells have been suggested in their reactivity to variants [8–10]. Plasma cells are highly specific to homologous antigens with high affinity, while B_mem_ cells tend to have BCRs with low affinity but relatively broad reactivity to heterologous antigens of variants [11–13].

In-depth immune profiling on SARS-CoV-2 convalescent and vaccinated individuals have greatly advanced our understanding of humoral immunity in relation to the breadth to virus variants. In this review, we discuss the recent progress in humoral memory responses to SARS-CoV-2. A comprehensive view of these responses will provide more insights into developing vaccines for the effective and broad control of SARS-CoV-2 variants that are likely to emerge in the future.

## Main text

### Neutralizing antibodies as an immune correlate of protection

**Fig. 2:**
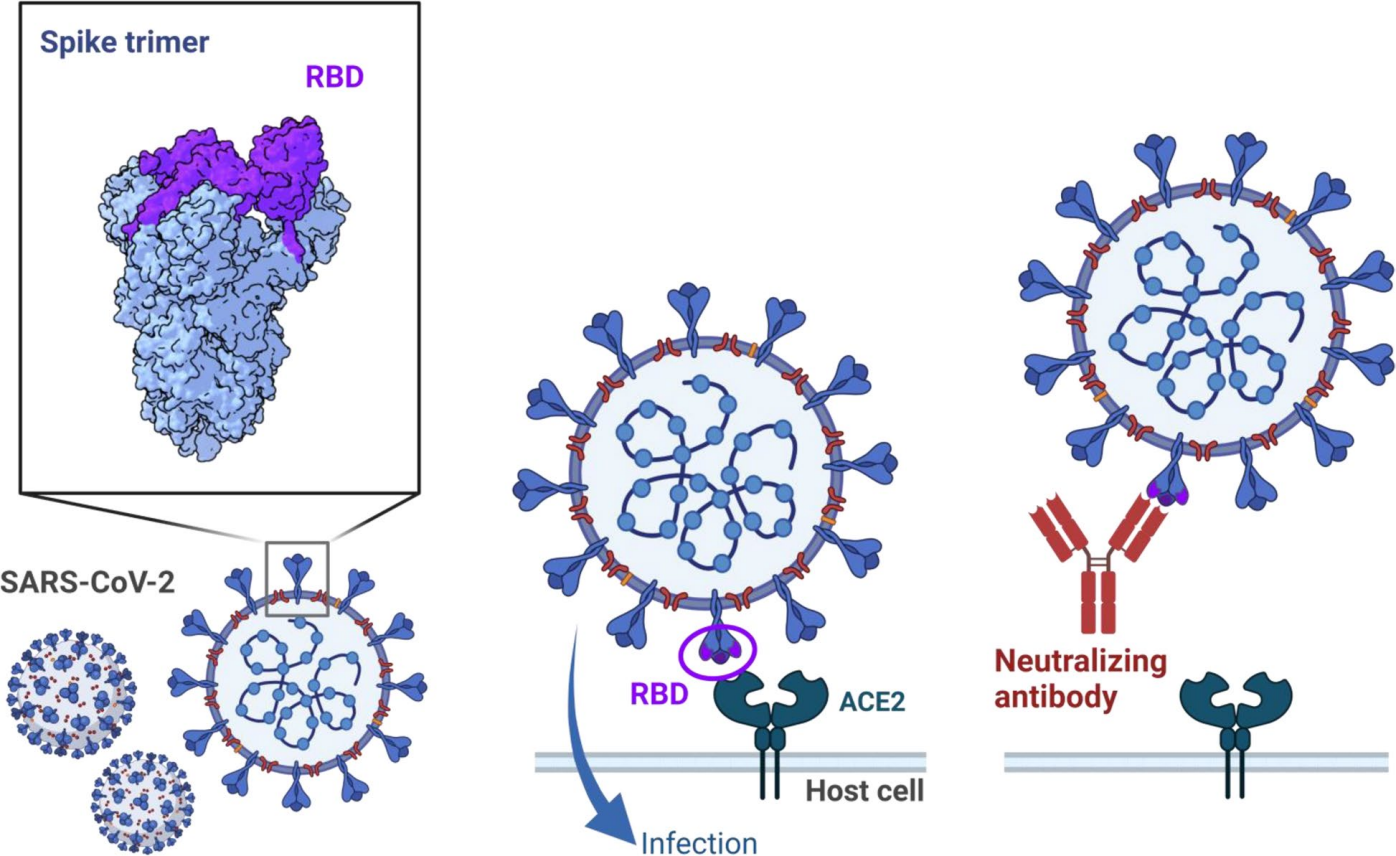
Antibody-dependent neutralization of SARS-CoV-2 entry through RBD. SARS-CoV-2 infection through RBD of spike proteins and neutralization by a protective antibody are depicted. The structural analysis on trimeric spike protein (upper left) is visualized with UCSF ChimeraX software (Refs. [35, 36]) using data from PDB (PDB ID: 6VSB, https://doi.org/10.2210/pdb6VSB/pdb, Ref. [37]). The figure was created with BioRender.com

SARS-CoV-2 contains structural proteins, including spike, envelope, membrane, and nucleocapsid proteins, and non-structural proteins encoded by several open reading frames [14]. The spike protein binds to host angiotensin-converting enzyme 2 (ACE2) through a receptor-binding domain (RBD), which is critical for viral entry into host cells [15–18] (Fig. 2). Although SARS-CoV-2 infection elicits antibodies targeting a variety of viral proteins, including structural and non-structural proteins [19], antibodies against spike RBD are particularly important for protective immunity, as they exhibit potent neutralizing activity [20–26]. Despite detection of neutralizing activity by non-RBD antibodies [27, 28], the majority of neutralizing antibodies circulating in the blood are known to target RBD [23–25]. The fact that all monoclonal antibody therapeutics in licensure recognize RBD epitopes [29–31] also illustrates the immunodominant nature of RBD for neutralizing antibodies. Furthermore, the successful use of neutralizing antibodies as an immune correlate of protection in vaccinees supports the functional role in preventing symptomatic diseases [32–34].

### SARS-CoV-2 variants escape the neutralizing antibody with RBD mutations

SARS-CoV-2 continuously changes its genome through mutations or viral recombination, and some emerging variants have acquired the ability to escape from neutralizing antibodies [38]. Antibody neutralization is one of the driving forces for selecting viral escape mutations, as observed during viral incubation in vitro under the pressure of neutralizing plasma [39–41]. Virus evolution has also been demonstrated in convalescent plasma treatment of an immunocompromised patient with persistent SARS-CoV-2 infection [42].

Variants with increased transmissibility, virulence, or immune escape are threats to global public health. Multiple SARS-CoV-2 variants with mutations in their spike RBD have emerged since the ancestral strain was identified in December 2019. The latest Omicron variant, which was detected in November 2021, subsequently diverged into a number of sublineages, e.g., BA.1, BA.5, BQ.1.1, and XBB. Each sublineage has 15 or more amino acid substitutions in its RBD [43]. The number of RBD mutations was greater than that of pre-Omicron variants with no more than three mutations. With numerous RBD mutations, Omicron variants strongly escape neutralizing antibodies [44–59]. A few antibodies retain neutralizing activity against a widely circulating Omicron subvariant, BA.5, by binding to conserved sites of the RBD between the ancestral and Omicron strains [57, 59].

### Temporal maturation of plasma antibodies induced by SARS-CoV-2 infection or vaccination

Serum antibodies induced by infection or vaccination act as the first wall of defense against future SARS-CoV-2 infections. Neutralizing antibodies of COVID-19 convalescent plasma peaks around 1–2 months after the infection, followed by a biphasic decrease, namely, early rapid (< 6 months), and late gradual (< 12 months) [60–62], roughly compatible with the kinetics of an influenza vaccine [5]. The titer declines but is still detectable even at late time points (5–12 months after infection). Indeed, spike-specific plasma cells are also detected in the bone marrow of convalescent individuals 11 months after infection [62].

The antibody titers induced by COVID-19 vaccination have essentially the same kinetics. COVID-19 vaccines have been designed to activate B cells specific to the SARS-CoV-2 spike, as the spike is a target of protective antibodies, as described above. Several modalities of COVID-19 vaccines have been developed, such as mRNA, viral-vector, inactivated whole virus, and protein vaccines. Among these, immune responses induced by mRNA vaccines, which were approved for human use for the first time, have been intensely analyzed. Vaccination induces neutralizing antibodies to SARS-CoV-2 in the plasma, which reaches its peak at approximately 1 month after the 2nd vaccination and then declines. The neutralizing activity is low but still detectable 5 months after the 2nd dose [63–66]. In line with this, spike-specific plasma cells can be detected at 5 months after vaccination in the draining lymph nodes and bone marrow [67]. The antibody response induced by an influenza vaccine persists up to 5 years above detectable levels [68]; however, it remains unclear how long the neutralizing activity induced by COVID-19 vaccine would remain above detectable levels.

Although the antibody quantity decreases, plasma antibodies mature in terms of neutralizing potency and breadth over time. Neutralization potency, which has been reported to correlate with the prognosis of patients [69], increased from the early to the late time points in convalescent plasma [61] and vaccinated plasma (unpublished data). Interestingly, neutralizing breadth to the SARS-CoV-2 variants also increases over time, despite individuals being exposed only to the ancestral strain [60, 61, 63, 65, 66]. The qualitative maturation of plasma antibodies after antigen exposure suggests gradual changes in antibody repertoires in long-lived plasma cells, which continue to replenish circulating antibodies over time [67].

### Repeated antigen exposures further improve humoral immune responses to the Omicron variants

Although plasma antibodies mature as mentioned above, the Omicron variants strongly escape vaccine-induced or convalescent serum polyclonal antibodies [44–48, 51, 53, 58, 59, 65, 70–77]. In some vaccinees, the neutralization titer for the Omicron variants after the 2nd dose of vaccination is not detectable throughout the time course. In other vaccinees with detectable titers against the Omicron variants, the Omicron neutralization titer after two doses of the vaccine has been reported to be 10 to > 120 times lower than that of the ancestral Wuhan strain.

Meanwhile, current studies on vaccinated cohorts have revealed that humoral immunity improves reactivity to Omicron variants even after the 3rd vaccination by the ancestral Wuhan-based strain [45, 46, 48, 77–79]. Neutralization titers to the Omicron variants after the 3rd dose of vaccines are still 5–10 times lower than those to the ancestral strain, but the difference is smaller than those after the 2nd dose from the same cohort. Improved neutralization to the Omicron variants has also been observed in breakthrough infections of pre-Omicron strains after two doses of vaccine [80]. Collectively, the three exposures to non-Omicron antigens induce neutralizing antibodies to the Omicron variants, indicating that repeated antigen exposure, even upon the ancestral strain, expands the breadth to the variants.

### Memory B cells are durably maintained with the breadth to variants

Exposure to the 3rd antigen with vaccination or breakthrough infection boosts the neutralizing titer to the same or higher level compared with the 2nd vaccination [45, 46, 48, 77–82]. Antibodies are largely supplied by plasma cells that originate from reactivated B_mem_ cells. As observed in influenza vaccine studies [6, 7], B_mem_ cells specific to the spike are stably maintained or even increase in number from 1 to 6 months after the 2nd vaccination [63–65]. These B_mem_ cell numbers further increase after the 3rd vaccination [79, 81].

B_mem_ cells exhibit extended cross-reactivity to the virus variants compared to antibodies secreted by plasma cells. Even after the 2nd dose of the vaccine, 30–50% of Wuhan RBD-reactive B_mem_ cells are cross-reactive to the Omicron variants, and their numbers increase over time [65, 81, 83]. A fraction of the Omicron-reactive B_mem_ cells retain BCRs which neutralize Omicron variants [65, 79, 83]. Thus, the 3rd vaccination and breakthrough infection reactivate the Omicron-neutralizing B_mem_ cells, which differentiate into plasma cells secreting Omicron-neutralizing antibodies. Indeed, a fraction of Omicron-reactive B_mem_ cells exhibit an activated phenotype after the 3rd dose [81].

The interval between antigen exposures is also a key factor in the cross-reactivity of humoral immune responses. A long interval between the 1st and 2nd doses of COVID-19 mRNA vaccines enhances the plasma neutralization titer against the Alpha, Beta, Gamma, and Delta variants compared with a short interval [84, 85]. At the breakthrough infection of non-Omicron variants after the 2nd vaccination, a longer interval from the vaccination induced a higher neutralization titer of the Omicron variant [80]. The longer interval may enable B_mem_ cells to undergo maturation for a longer time to increase cross-reactivity before reactivation.

**Fig. 3:**
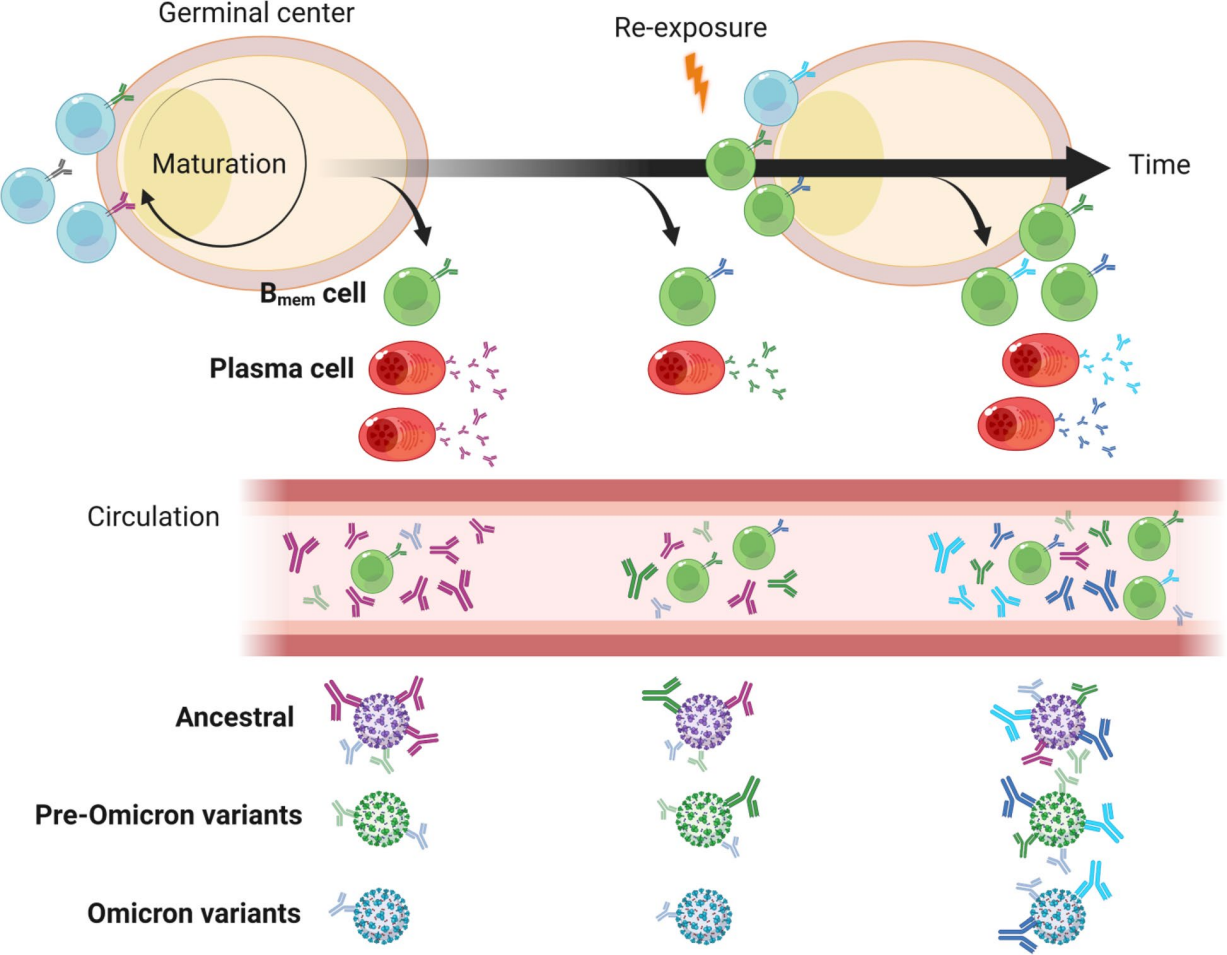
Evolution of humoral immune responses to SARS-CoV-2. B_mem_ cells evolve their BCRs over time to acquire the breadth to pre-Omicron variants. The BCR breadth is further expanded to Omicron variants after re-exposures by vaccination or infection. These processes may play a pivotal role in durable and broad protection against SARS-CoV-2 variants that emerge in the future. The figure was created with BioRender.com

Collectively, B_mem_ cells have evolved their BCR repertoire to acquire broad reactivity to the variants over time after ancestral strain-based vaccination (Fig. 3). Hence, B_mem_ cells may play a pivotal role in both the durability and breadth of protection against emerging SARS-CoV-2 variants.

### Memory B cell selection underlying the evolution

B_mem_ cells encompass phenotypically and functionally heterogeneous subsets [3, 86, 87]. After the 2nd vaccination, the subset composition of B_mem_ cells changes over time, skewing toward CD21^+^CD27^+^ resting B_mem_ cell dominance from the early to late time points [65]. The accumulation of resting B_mem_ cells is due to an increase in the number of cross-reactive resting B_mem_ cells but not due to the B_mem_ cells that are reactive only to the ancestral strain, resulting in the evolution of reactivity in the resting B_mem_ cells. CD27^+^ B_mem_ cells, including the resting subset, are intrinsically programmed to enhance survival and rapidly respond to restimulation [88]. Hence, the increase in the evolved cross-reactive resting B_mem_ cells may contribute to a rapid cross-reactive antibody response after antigen re-exposure, as observed at the 3rd vaccination [81].

Antigen exposure, such as infection or vaccination, shapes the B cell repertoire through BCR-dependent selection and maturation. The antigen activates antigen-reactive naïve B cells and B_mem_ cells, which in turn undergo B_mem_/plasma cell differentiation or further maturation in GCs in lymphoid tissues, where B cells diversify their BCRs and undergo selection [8, 89–91]. Throughout the GC reaction, a fraction of GC B cells differentiate into B_mem_ and plasma cells. These processes diversify and continuously change the BCR repertoire, which may contribute to the evolution of B_mem_ and plasma cell BCRs induced by SARS-CoV-2 infection and vaccination. Indeed, GCs have been detected in the draining lymph nodes of mRNA vaccinees and persist for months [67, 92, 93]. The B_mem_ cells after vaccination accumulate mutations in their BCRs over time [63, 79], indicating that the vaccine-induced B_mem_ cells are derived from the GCs. In addition, COVID-19 convalescent individuals also sustain B_mem_ cells with mutated BCRs and bone marrow plasma cells, which is indicative of their GC-dependent development [60, 62, 94]. Although patients with severe COVID-19 harbor dysfunctional responses at acute phase [95, 96], the higher number of circulating follicular helper T cells and comparable affinity maturation of plasma IgG titer in the convalescent phases suggest the involvement of functional GC responses at a different time or location [97, 98]. Otherwise, non-GC pathway could be involved in severe cases [96]. B_mem_ cell evolution may involve B-cell selection in GCs over time. The prolonged interval between antigen exposures may result in a longer selection of B_mem_ cells before reactivation, which in turn confers more evolved humoral antibody responses, as mentioned above. In COVID-19 convalescent individuals, B_mem_ cells also evolve their reactivity against variant strains [60, 99]. The evolved cross-reactivity of a B_mem_ cell pool can be achieved in two ways: mutation-dependent maturation of pre-existing B_mem_ cell clones, and expansion of newly emerging clones with cross-reactivity. B_mem_ cells after the 3rd vaccination include both persistent and newly emerged B_mem_ cell clones [79], indicating the contribution of both pre-existing and new B_mem_ cells to the evolving reactivity of the BCR repertoire. The BCRs of the newly emerging clones bind distinct epitopes from those of the other clones, thereby changing the epitope distribution of the overall B_mem_ cell repertoire.

One possible mechanism underlying the change in paratope distribution is a process called antibody feedback. In this process, the antibodies in circulation mask the antigenic epitopes for B-cell selection and suppress B cell activation toward the same epitopes [100–104]. COVID-19 vaccines or active SARS-CoV-2 infection predominantly activate spike-specific B cells toward immunodominant epitopes [23, 105]. Activated B cells expand and differentiate into GC B, B_mem_, and plasma cells, which in turn secrete antibodies. The predominant antibodies produced at the early stage may dampen the selection of B cells specific to the predominant epitopes at the later stage and the next vaccination, resulting in a change in the predominant epitopes of the B cell repertoire over time. A study demonstrated that antibody feedback by monoclonal antibodies alters epitopes of B_mem_ cells induced by COVID-19 vaccination [106].

Whereas repeated COVID-19 vaccination improves cross-reactivity to variant strains, repeated vaccination with homologous flu hemagglutinin (HA) skews antibody response towards the more specific HA head domain of the targeted strain [107]. Concordantly, compared to plasmablasts, a single dose of HA vaccination induces GC B cells with more specific reactivity against the vaccinated HA, which may be involved in later humoral responses [108]. Some of those GC B cells recognize different epitopes on the HA from those plasma antibodies bind. Thus, homologous flu HA protein and COVID-19 mRNA vaccines both induce the evolution of B cell responses, but to opposite directions, specific and broad reactivity, respectively. The discrepancy in the direction may be related to the properties of cross-reactive antibodies and their epitopes, since most of flu cross-reactive antibodies which target well-conserved stalk region of HA are polyreactive [107]. This implies a selective disadvantage of the flu cross-reactive B cells due to immune tolerance. Collectively, the B_mem_ cell evolution is induced independent of antigens and modality, while the direction of the evolution may vary by antigens.

Further investigation of the mechanism underlying the evolution of broadly reactive B_mem_ cells, induced by COVID-19 vaccination, is indispensable for the development of universal vaccines that confer durable and broad protection against future SARS-CoV-2 variants and other pandemic viruses.

## Conclusion

The repeated emergence of SARS-CoV-2 variants escaping vaccine-induced immune responses has emphasized the importance of developing universal vaccines that induce protective immune memory toward a variety of variants, ideally including future escaping variants. Recent intense cohort studies on COVID-19-convalescent and vaccinated individuals have revealed that B_mem_ cells play a pivotal role in durable and broad humoral memory responses. B_mem_ cells evolve their reactivity to the variants over time, which are reactivated to secrete antibodies into circulation in response to subsequent antigen exposure. In summary, persistent maturation and repeated reactivation are the two key events involved in the evolution of humoral immune responses to SARS CoV-2. Although most COVID-19 vaccine studies have focused on responses to mRNA vaccines, some studies have reported that vaccines with other modalities induce B_mem_ cells of similar quality [109, 110]. In addition to these modalities, switching to Omicron-adapted vaccines from the ancestral strain is considered and discussed in the current situation of the Omicron outbreak. However, the cross-reactivity of B_mem_ cells induced by the Omicron-adapted vaccine booster is comparable to that of the ancestral booster in nonhuman primates [111]. Further analyses of B-cell responses to vaccines with various modalities and antigens will provide more insights into the evolution of B_mem_ cells, which may be pivotal for the development of universal vaccines.

## Data Availability

Not applicable.

## Abbreviations

ACE2: Angiotensin-converting enzyme 2
BCR: B-cell antigen receptor
B_mem_ Cell: Memory B cell
COVID-19: Coronavirus disease 2019
GC: Germinal center
HA: Hemagglutinin
RBD: Receptor-binding domain
SARS-CoV-2: Severe acute respiratory syndrome coronavirus 2
